# In vitro effects of 0 to 120 Grays of irradiation on bone viability and release of growth factors

**DOI:** 10.1186/s12903-016-0241-9

**Published:** 2016-07-04

**Authors:** Kosaku Sawada, Masako Fujioka-Kobayashi, Eizaburo Kobayashi, Jens O. Brömme, Benoit Schaller, Richard J. Miron

**Affiliations:** Department of Cranio Maxillofacial Surgery, Inselspital, University of Bern, Bern, Switzerland; The Nippon Dental University, School of Life Dentistry at Niigata, Advanced Research Center, Niigata, Japan; Department of Radiation Oncology, Inselspital, University of Bern, Bern, Switzerland; Department of Oral Surgery and Stomatology, Department of Periodontology, University of Bern, Bern, Switzerland; Department of Periodontology, College of Dental Medicine, Nova Southeastern University, 3200 South University Drive, Fort Lauderdale, Florida 33328 USA

**Keywords:** Irradiation, Bone cell death, Grays, Growth factor release, Bone chips

## Abstract

**Background:**

High dose radiation therapy is commonly used in maxillofacial surgeries to treat a number of head and neck tumors. Despite its widespread use, little information is available regarding the effects of irradiation on bone cell viability and release of growth factors following dose-dependent irradiation.

**Methods:**

Bone samples were collected from porcine mandibular cortical bone and irradiated at doses of 0, 7.5, 15, 30, 60 and 120 Grays. Thereafter, cell viability was quantified, and the release of growth factors including TGFβ1, BMP2, VEGF, IL1β and RANKL were investigated over time.

**Results:**

It was observed that at only 7.5Gy of irradiation, over 85 % of cells were non-vital and by 60 Gy, all cells underwent apoptosis. Furthermore, over a 7-fold decrease in VEGF and a 2-fold decrease in TGFβ1 were observed following irradiation at all tested doses. Little change was observed for BMP2 and IL1β whereas RANKL was significantly increased for all irradiated samples.

**Conclusions:**

These results demonstrate the pronounced effects of irradiation on bone-cell vitality and subsequent release of growth factors. Interestingly, the largest observed change in gene expression was the 7-fold decrease in VEGF protein following irradiation. Future research aimed at improving our understanding of bone following irradiation is necessary to further improve future clinical treatments.

## Background

High doses of irradiation therapy are routinely administered to patients for a large number of cancers affecting many organs [1, 2]. Furthermore, the use of bone allografts necessitates high dose irradiation for sample sterilization [1, 2]. Under normal circumstances, irradiation passes through a number of tissues where the minimization of doses is vital to the future survival of tissues. One tissue that commonly receives high doses of irradiation therapy in maxillofacial surgery is that of bone. Due to the high volume of bones found in the maxilla, it is common in head and neck procedures to pass high doses of irradiation through bony tissues [1, 2]. Common doses can range from 60 Gray to 70 Grays and such procedures have been highly successful for the treatment of many head and neck tumors [3, 4]. Despite successfully treating and managing these tumors, irradiation of bone has reported some drawbacks with a certain percentage of bone losing complete vitality and becoming necrotic [3, 4]. A group of experts have since recommended guidelines established for preventing the possibility of developing osteoradionecrosis of the jaw at doses exceeding 60 Grays [3, 4].

Furthermore, irradiation of bone is also commonly found in bone allograft sterilization procedures for bone tissue banking [5]. Although the full set of doses are commonly kept proprietary information [5], the release of subsequent growth factors from its content is a trademark commonly observed in demineralized bone allografts which may demonstrate signs of osteoinduction by showing signs of ectopic bone formation in various animal models. Irradiation of bone is also used in extracorporeal irradiation and has been implicates in not only oral and maxillofacial surgery, but also other disciplines such as otolaryngology [6] and orthopedics [7].

Marx et al. have been key oral maxillo-facial surgeons responsible for treating and developing treatment guidelines for patients presenting necrotic bone following irradiation [8–10]. While a number of attempts have been investigated to maintain optimal bone viability [11–13], a limited understanding of the cellular events that take place within bone following irradiation would benefit from further investigation.

Due to the widespread use of irradiation for various bone procedures including irradiation for tumors, sterilization of autologous bone transplants and bone tissue banking, it became of interest to our group to further investigate in a bench model the effects of dose-dependent irradiation on bone cell viability and release of growth factors. While it is known that irradiation is unfavorable for bone remodeling [14] and that an accumulation of evidence has been accumulating demonstrating that irradiation consequently affects microvasculature of bone-tissues [15], a detailed investigation of the in vitro mechanisms was studied to further increase our understanding of bone changes following irradiation. Therefore, the purpose of the present investigation was to determine the effects of irradiation on bone cell viability following irradiation at various single-doses including 0, 7.5, 15, 30, 60 and 120 Grays. Thereafter, bone graft morphology and surface proteins were analyzed via scanning electron microscopy and release of growth factors from the bone samples was quantified for TGFβ1, BMP2, VEGF, IL1β and RANKL at 15 min and 4 h following irradiation.

## Methods

### Bone collection

Bone was obtained from adult pigs (Metzgerei Balsiger, Wattenwil, Switzerland) and harvested from the buccal-sided mandibular cortical bone with a “bone scraper” (Hu-Friedy, Rotterdam, Netherlands) and placed into sterile plastic dishes as previously described [16]. Thereafter, bone was irradiated (single-dose) at the following doses: 0 (control), 7.5, 15, 30, 60 and 120 Grays. Briefly, bone samples were collected and exposed to ^137^Cs γ-rays at the dose rate of 0.83 Gy/min using a Gammacell®40 Exactor (Best Theratronics, Ottawa, Canada) for 0, 9, 18, 36, 72 and 144 min until the final dose of irradiation was reached. Thereafter, bone samples were immediately placed in a cell culture hood and experiments performed. For each experiment, four independent preparations of bone samples were available and all samples were performed in triplicate. Thereafter bone samples were either 1) fixed and assigned to scanning electron microscopy, 2) assigned for MTS analysis for cell viability or 3) left in PBS solution and samples collected after 15 min and 4 h for protein quantification using ELISA.

### Scanning electron microscopy

Bone samples were fixed in 1 % glutaraldehyde and 1 % formaldehyde for 2 days for scanning electron microscopy (SEM). Following serial dehydration with ethanol, samples were critical point dried (Type M.9202 Critical Point Dryer, Roth & Co. Hatfield, PA, USA) and allowed to dry overnight as previously described [17, 18]. The following day, samples were sputter coated using a Balzers Union Sputtering Device (DCM-010, Balzers, Liechtenstein) with 10 nm of gold and analyzed microscopically using a Philips XL30 FEG scanning electron microscope to determine surface variations between samples.

### Quantification of viable cells in bone samples

The cell viability in each of the bone samples was determined using the CellTiter 96® One Solution Cell Assay (MTS) (Promega, Madison, WI, USA) as previously described [19]. Briefly, 100 mg of harvested bone was treated with 80 μL of CellTiter96 aqueous solution dissolved in 400 μL of PBS. After 4 h of incubation, the cell viability was determined by measuring the absorbance at 490 nm on a 96-well plate-reader. Experiments were performed in triplicate with three independent experiments for each condition. Data was normalized to samples at 120 Gray with no signs of cell viability and analyzed for statistical significance using one-way analysis of variance with Tukey’s test.

### ELISA protein quantification

Specific protein contents were determined for cell culture media incubated with 250 mg of bone samples. At time points 15 min and 4 h BMP2, TGFβ1, VEGF, IL1β and RANKL were quantified using an ELISA assays (RND Systems, Minneapolis, MN, USA) according to manufacturer’s protocol as previously described [17, 20]. Briefly, 100 μl of assay diluents and 50 μl of sample were incubated for 2 h at room temperature in antibody-precoated 96-well plates. Wells were washed 4 times with washing buffer, incubated for 2 h with peroxidase-conjugated antibody solution, washed again, followed by addition of 200 μl of substrate solution for 30 min and 50 μl of stopping solution for 30 min. Absorbance was measured at 450 nm on an Infinite 200 microplate reader (Tecan Group LTD, Männedorf, Switzerland). All samples were measured in triplicate and 3 independent experiments were performed. Statistical analysis was performed by two-way ANOVA with Bonferroni test.

## Results

### Scanning electron microscopy

**Fig. 1:**
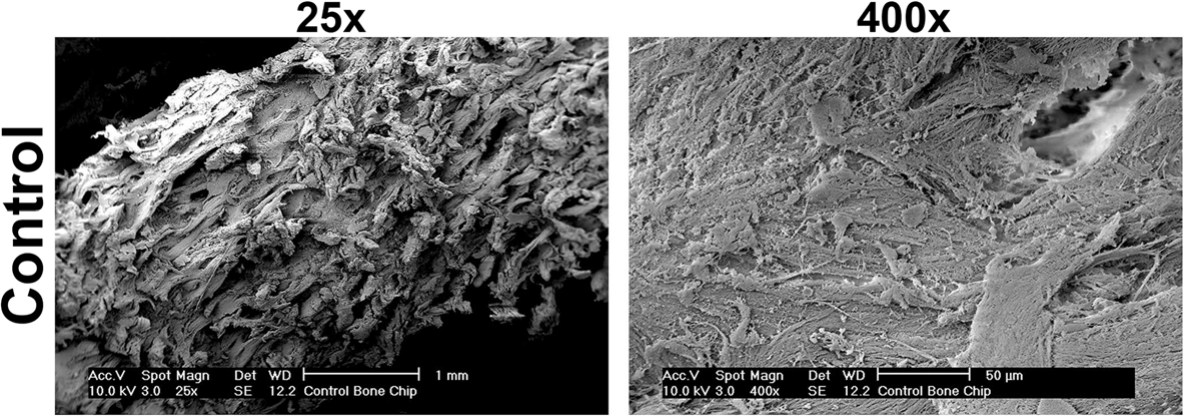
Scanning electron micrograph (SEM) of control bone samples at magnifications of both 25 times and 400 times. Note the visible protein content found on the surface of bone at a 400 times magnification

**Fig. 2:**
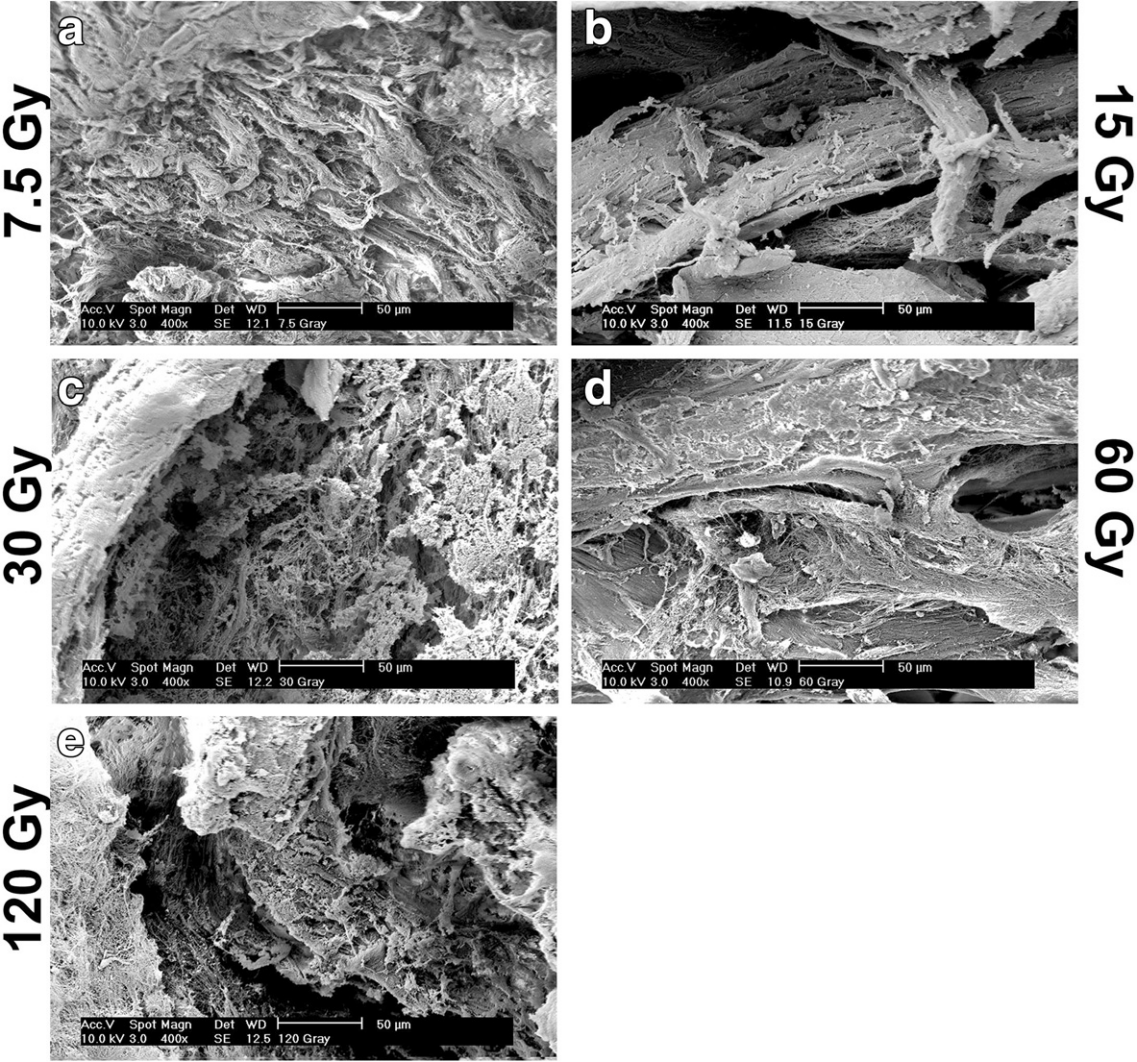
Scanning electron micrograph (SEM) of irradiated bone samples at a magnification of 400 times for (**a**) 7.5, (**b**) 15, (**c**) 30, (**d**) 60 and (**e**) 120 Grays. Following increasing concentrations of irradiation, little to no changes in the surface homogeneity of proteins could be observed across all irradiated samples

Bone samples were first visualized by SEM for morphologic differences before and after irradiation (Figs. 1, 2). Control samples were first utilized to determine surface characteristics prior to irradiation (Fig. 1). It was observed at 25 times magnification that bone samples presented many roughened topographies (Fig. 1). At higher resolution (400x magnification), the surface of control bone samples demonstrated many visible proteins on the surface of bone samples with a homogeneous surface coating of proteins typically found in native bone (Fig. 1). Following irradiation, bone samples visualized by SEM demonstrated a very homogeneous layer of surface proteins remaining on the bone surface (Fig. 2). Under the present conditions, very little change could be observed for bone samples at all doses with a common protein layer found across all surfaces independent of irradiation doses up to 120 Gy (Fig. 2).

### Cell viability within bone samples

**Fig. 3:**
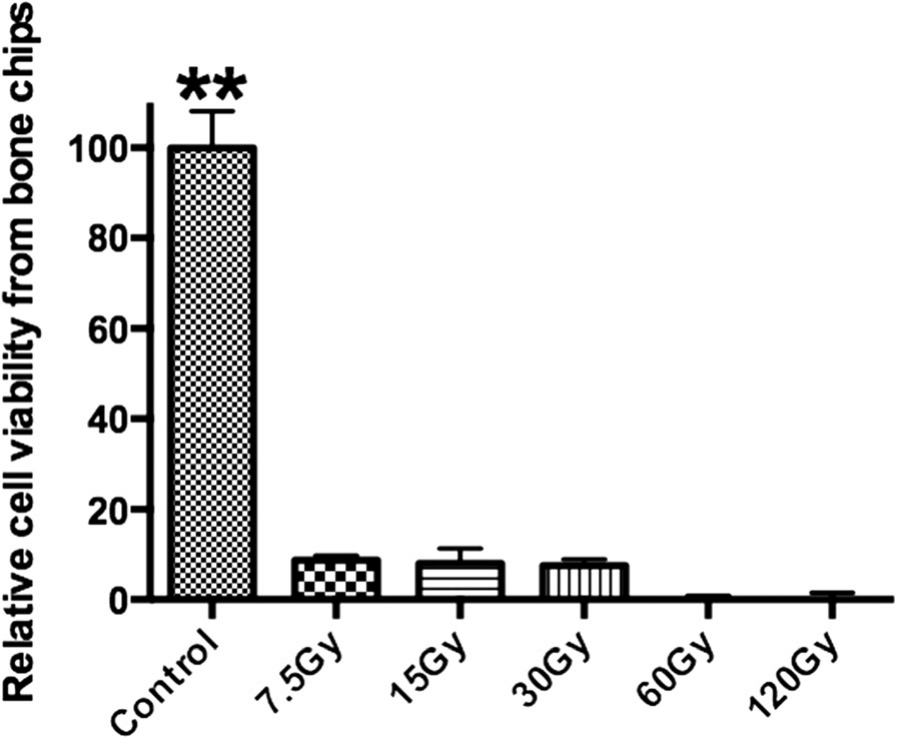
Relative cell viability of bone cells following irradiation at varying concentrations of Grays relative to control samples. Over 85 % of all cells were non-vital in samples receiving as little as 7.5 Gy and all cells were non-vital following 60 Gy. (** denotes significantly higher than all other treatment modalities *p <* 0.01)

Bone samples were quantified by MTS assay in order to determine the amount of living bone cells found within samples following irradiation at doses ranging from 0 to 120 Gy. After only 7.5 Gy of irradiation, it was observed that over 85 % of all cells found within bone samples were non-vital (Fig. 3). After 60 Gy of irradiation, it was found that 0 % of cells were still viable (Fig. 3). The results from this experiment confirm the extremely damaging and significant effect of even small doses of irradiation on vitality of cells found within the bone matrix (Fig. 3).

### Release of growth factors from within bone samples

**Fig. 4:**
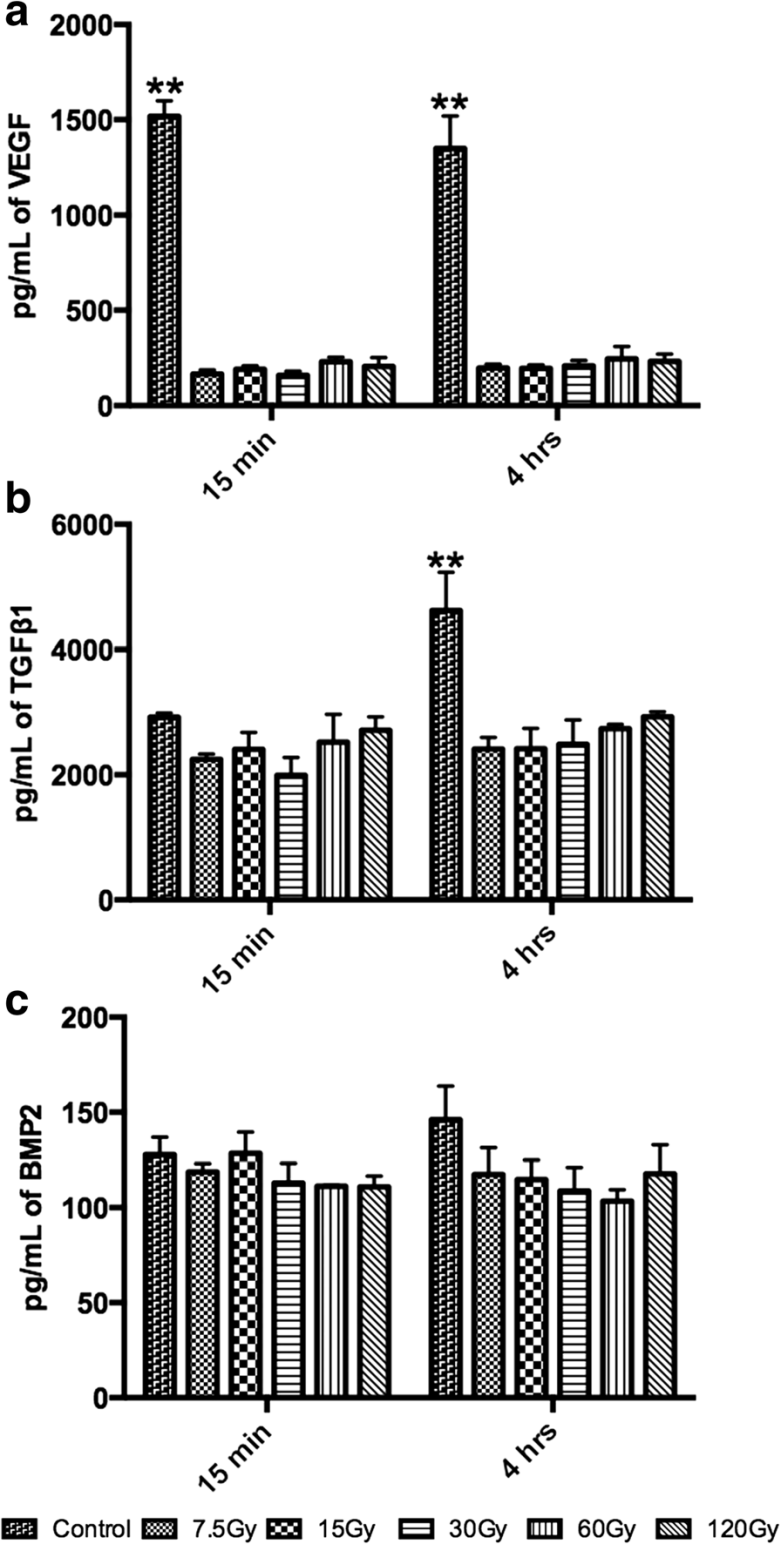
Elisa quantification for growth factors including (**a**) vascular endothelial growth factor (VEGF), (**b**) transforming growth factor beta 1 (TGFB1) and (**c**) bone morphogenetic protein 2 (BMP2). **a** Following irradiation at all doses, a marked and significant decrease in VEGF was noted at both 15 min and 4 h. **b** Similarly, TGFB1 had a significant 2 fold decrease at 4 h for irradiated samples. **c** No changes in BMP2 could be observed at all time points following irradiation. (** denotes significantly higher than all other treatment modalities *p <* 0.01)

**Fig. 5:**
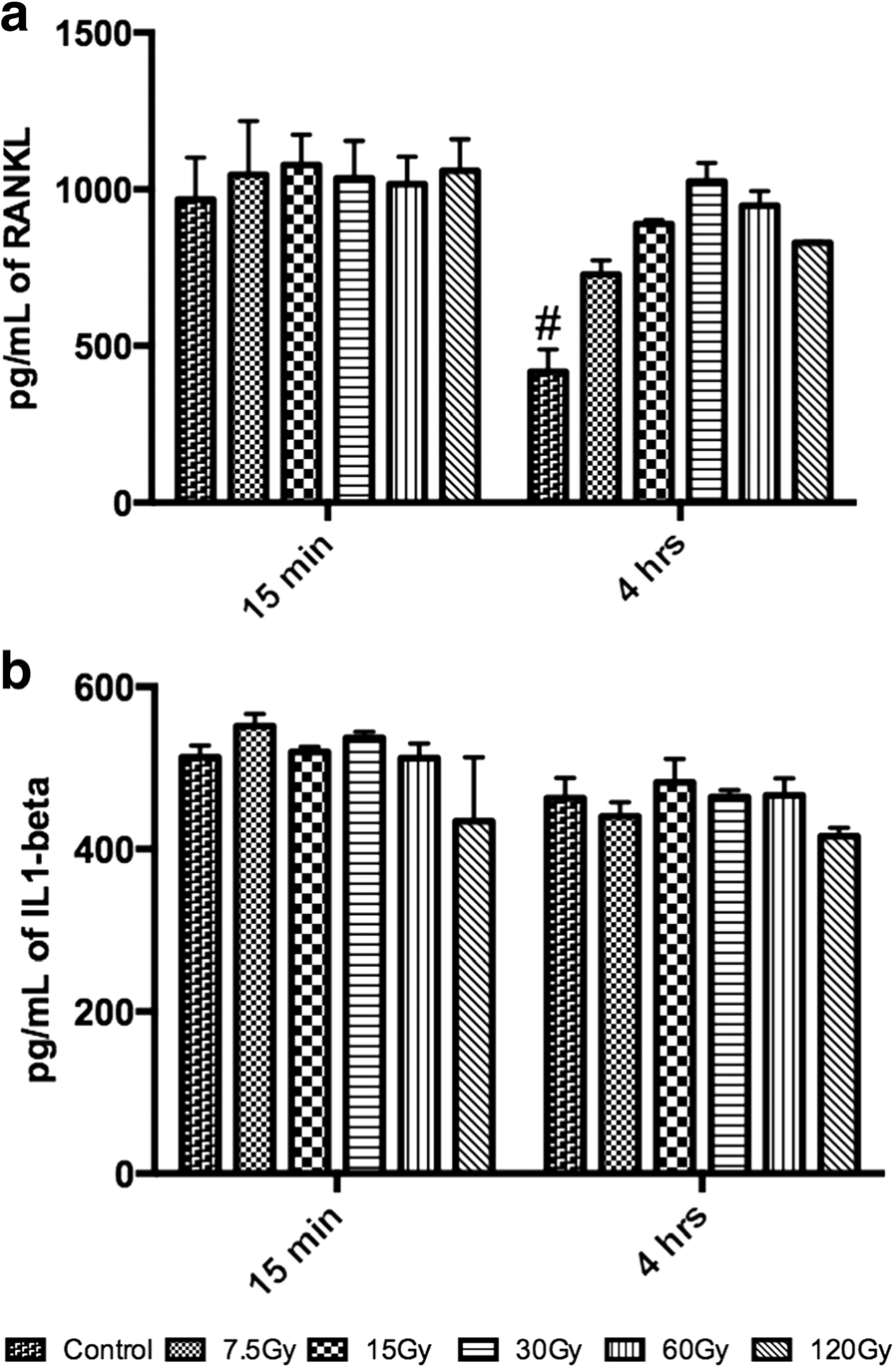
Elisa quantification for growth factors including (**a**) Receptor activator of nuclear factor kappa-B ligand (RANKL) and (**b**) interleukin 1-beta (IL1B). **a** Following irradiation at 4 h, a 2 fold decrease in RANKL could be observed for control samples when compared to all irradiated doses. **b** No changes in IL1B could be observed at all time points following irradiation. (# denotes significantly lower than all other treatment modalities *p <* 0.01)

All bone samples were then quantified for release of growth factors including VEGF, TGFβ1, BMP2, RANKL and IL1β following 15 min and 4 h (Fig. 4, 5). It was first found that a high concentration of VEGF of 1517 +/- 142 pg/mL could be observed for control samples at 15 min and this was relatively maintained following 4 h of sample preparation (Fig. 4a). Interestingly, a marked and pronounced significant decrease of VEGF was observed in all irradiated bone following as little as 7.5 Gy of irradiation (Fig. 4a). No significant differences could be observed between all samples irradiated with bone at either 15 min or 4 h (Fig. 4a). Then TGFβ1 was quantified and although no significant differences could be observed at 15 min post irradiation, approximately a 2 fold significant increase was observed for control samples at 4 h (4621 +/- 1058 pg/mL). Interestingly, no differences in BMP2 protein concentration could be observed at both time points with very little expression observed in all samples (control samples = 127 +/- 15 pg/mL at 15 min and 146 +/- 30 pg/mL at 4 h).

Following growth factor concentration analysis, IL1β and RANKL were then quantified for the effects of bone irradiation on inflammatory cytokines and osteoclast differentiation marker (Fig. 5). First it was found that at 15 min post irradiation, no differences in RANKL expression could be observed between control samples and all irradiated bone samples (Fig. 5a). By 4 h, a significantly lower expression of RANKL could be observed when compared to all irradiated bone samples although all results were less than a 2 fold significant increase (Fig. 5a). No differences could be observed between all samples at either time points for IL1β expression (Fig. 5b).

## Discussion

The purpose of the present manuscript was to determine in detail the cellular events that occur following irradiation of bone samples at increasing doses. Although clinically doses can range in intensity and duration, it has been suggested in the literature that doses exceeding 60 Gy are more commonly associated with osteoradionecrosis of the jaw [3, 4]. Furthermore, a number radiation therapy complications have been reported in the literature to date [21]. Therefore, the aim of this bench top study was to perform an in vitro investigation to further understand the cellular events taking place within bone samples following irradiation.

Interestingly, in the present study very little change in surface proteins was found between all bone samples following irradiation (Figs. 1, 2). Furthermore, it was observed that in all samples treated with irradiation, over an 85 % cell mortality was seen even following as little as 7.5 Gy (Fig. 3). This result was extremely surprising as it was initially thought that such small doses would have very little effect on cellular viability. It must be noted that this reported finding applies solely to an in vitro model and its extrapolation to the clinical reality is limited given that immune cells and regenerative cells would minimize free radicals and cells death when compared to the present in vitro model. Nevertheless, it remains striking that cell death occurred in such high numbers following such low levels of irradiation doses.

Thereafter, samples were investigated for growth factor and cytokine release following irradiation at both 15 min and 4 h post irradiation. The reason for selecting these time points was specifically to investigate the changes in cytokine release after a short time interval following initial irradiation (15 min) and also to determine how release of cytokines was affected after a later time point (4 h) following cell death from irradiation. The bone samples were first assessed for VEGF protein release. There was a marked and significant decrease in release of VEGF as early as 15 min post-irradiation (Fig. 4a). VEGF is one of the key growth factors responsible for angiogenesis and the effects of irradiation demonstrate the harsh effects on this potent growth factor. The results from this study further demonstrate and support the groups of clinical experts working with oxygen delivered in hyperbaric pressures for improved angiogenesis before and/or after irradiation therapy [11–13]. Although the clinical efficacy of using such treatment has come under speculation in recent years [22], the rational behind improving the angiogenic properties of bone for irradiated patients is logical and can further be explained by the present study as VEGF was the growth factor most notably down-regulated following irradiation (Fig. 4a). Future research aimed at addressing the in vivo release of VEGF from bone following irradiation may further add valuable data supporting the findings from the present study that irradiation has a significantly pronounced effect on VEGF protein release following irradiation at various doses.

Interestingly, it has been shown in a recent report that osteocytes are major contributors to the release of VEGF in vivo [23]. Furthermore, a subsequent in vitro report has demonstrated that proton irradiation with as little as 2 Gy is enough to suppress angiogenic genes in certain cell types [24]. Taken together with the results from the present study, it becomes extremely clinically relevant to further design strategies to limit the down-regulation of pro-angiogenic genes.

A second growth factor that has been extensively investigated by our group with respect to growth factor released from bone samples is TGFβ1 [17, 25–27]. In several studies analyzing the released protein content from bone (termed bone conditioned medium (BCM)), it was found that one of the likely paracrine factors displayed in bone remodeling is that of TGFβ1 [25]. It was found that by inhibiting TGFβ1 pathway, a 5-fold decrease in oral fibroblast activity was observed, thus confirming that much of the preliminary remodeling process caused by bone is likely governed by TGFβ1 signaling [27]. Thus, in the present study, a 2 fold significant decrease in TGFβ1 protein expression released from the bone samples following irradiation is likely to have a significant effect on future bone remodeling following irradiation. Furthermore, the combinatory reduction of both TGFβ1 and VEGF is hypothesized to pose major bone remodeling challenges further illustrating the necessary regenerative procedure to counteract these major drawbacks.

It was surprisingly observed in the present study that irradiation had virtually no effect on BMP2 protein expression (Fig. 4c). It may therefore be concluded that dying cells that are found within the bone matrix are not target cells for release of osteoinductive growth factors such as BMP2. Interestingly, it is commonly reported in the literature that certain forms of demineralized freeze-dried bone allografts (DFDBA) are osteoinductive whereas most if not all non-demineralized samples are non-osteoinductive [28]. Therefore, it may be concluded that within the present investigation, BMP2 is not a key player in bone remodeling of irradiated bone and likely BMP2 expression is only upregulated once the bone samples are resorbed by osteoclasts and BMP2 is thereafter released from content coming from within the bone matrix.

The results obtained with RANKL protein quantification also generated statistically significant differences at 4 h between control and irradiated bone. RANKL protein expression was up to 2 fold lower than certain irradiated bone samples. It must once again be highlighted that the largest percentage of cells found within bone samples are osteocytes, which account for approximately 90 % of all bone cells. It has previously been demonstrated that damaged or lack of osteocytes is routinely associated with reduced remodeling [29, 30] and dying osteocytes are able to signal for bone resorption by attracting osteoclasts through the release of RANKL [31, 32]. The in vitro findings in the present experiment further demonstrate and confirm the ability for bone samples undergoing high and fast rates of cell death are able to release osteoclast differentiation marker RANKL to the surrounding environment following cell death.

It must also be considered that one of the study limitation of the present study were that all bone samples received irradiation directly to bone in an in vitro model which might not simulate a clinical situation. An in vivo model would necessitate that all irradiation to bone would ultimately pass through overlying tissue which include epithelial, connective tissues, glands, muscles and a combination of them therefore absorbing some of the irradiation prior to bone. Furthermore, immune cells would counteract some of the free radicals produced by irradiation likely contributing to apoptosis of various cell types. Future investigation characterizing this interplay between these various cell types would further contribute to our understanding of bone remodeling following irradiation. Furthermore, in the present model, the bone periosteum was removed which might have a significant influence of the final outcome. Many of the progenitor cells found within bone are located within the periosteum and this complex interaction between periosteum and bone requires better understanding to better implement future regenerative procedures.

In context with some of the known literature, it has been debated for some years the influence of osteoradionecrosis on bone cell interactions. The original proposed and well-accepted ‘three-H concept’ of hypoxia, hypocellularity and hypovascularity as defined by Marx brings into question all the key elements of bone viability [33]. In light of the present findings, it becomes apparent that one of the key components downregulated after irradiation is that of VEGF thus giving evidence for a hypovascular and hypoxic environment. We also demonstrate the drastic changes in cell viability following only 7.5 Gy of irradiated bone. Although these doses would be significantly different in a human model and that the present in vitro model can only be vaguely extrapolated to a clinical situation, it remains highly pertinent information that 2 of the most affected genes, VEGF and TGFβ1, are prominent growth factors for bone regeneration.

Furthermore, most of the accumulated evidence from this manuscript seems to suggest that it is the osteocytes that are playing a key role in this process following irradiation. As most of the cells are apoptotic following irradiation, it becomes evident that they are major key players in maintaining tissue vascularity as they are key players in VEGF production. In previous histologic studies, it was found in human specimen samples from osteoradionecrotic bone after 36 Gy, a loss of osteocytes could be observed [34, 35]. Thus, it remains essential to further study the relationship between irradiated bone and most specifically osteocytes. Future research aimed at investigating protein release of growth factors such as TGFβ1 and VEGF using an animal model would be extremely advantageous. A further understanding of this relationship could provide more pertinent information to clinicians to better gear regenerative procedures for the treatment of osteoradionecrosis of the jaw and possibly provide better preventative measures for these patients prior to complications.

## Conclusions

The analysis of bone samples following irradiation demonstrated quite profound effects on cell viability following irradiation and the release of growth factors responsible for bone regrowth and/or bone remodeling were also significantly affected. Over 85 % of cell death occurred following only 7.5 Gy of irradiation to bone samples and a 7-fold decrease of VEGF and a 2-fold decrease in TGFβ1 protein quantification were observed. Furthermore, RANKL was upregulated approximately 2 fold in samples receiving irradiation. As the effects of irradiation on bone viability and release of proteins was quite pronounced in the present study, it may thus becomes vital to better understanding the cell mechanisms taking place following irradiation. Future animal study investigating growth factor release from bone following irradiation would be beneficial.

## Abbreviations

TGFβ1, transforming growth factor Beta 1; BMP2, bone morphogenetic protein 2; VEGF, vascular endothelial growth factor; IL1β, interleukin 1 beta; RANKL, receptor activator of nuclear factor kappa-B ligand; PBS, phosphate buffered solution; ELISA, enzyme-Linked Immunosorbent assay; SEM, scanning electron microscopy; BCM, bone conditioned medium
